# Electrocardiogram-Alterations and Increasing Cardiac Enzymes After Coronary Artery Bypass Grafting—When Can We Expect Significant Findings in Coronary Angiography?

**DOI:** 10.3390/medicina61122192

**Published:** 2025-12-11

**Authors:** Ali Taghizadeh-Waghefi, Manuel Wilbring, Asen Petrov, Sebastian Arzt, Utz Kappert, Sems-Malte Tugtekin, Klaus Matschke, Konstantin Alexiou

**Affiliations:** 1 Medical Faculty “Carl Gustav Carus”, Technical University of Dresden, 01307 Dresden, Germany; 2 Center for Minimally Invasive Cardiac Surgery, University Heart Center Dresden, 01307 Dresden, Germany

**Keywords:** coronary artery bypass grafting, bypass surgery, perioperative myocardial infarction

## Abstract

*Background and Objectives*: Perioperative myocardial infarction (PMI) after coronary artery bypass grafting (CABG) remains difficult to diagnose due to varying biomarker thresholds and ECG criteria. This study aimed to evaluate the predictive value of ECG changes and cardiac biomarkers for identifying pathological findings in repeat coronary angiography after CABG. *Materials and Methods*: This retrospective study included 137 patients who underwent repeat coronary angiography due to suspected PMI. ECG changes and serial measurements of CK, CK-MB, and hsTnT were analyzed at 4, 8, and 18 h postoperatively. The primary endpoint was the identification of graft-related complications or new coronary lesions. *Results*: Pathological angiographic findings were detected in 85.4% (*n* = 117) of cases, predominantly graft-related complications (96.6%). ST-segment elevation (*p* < 0.01) and ST-segment depression (*p* = 0.02) were significantly associated with pathological findings. The combination of ST-segment elevation and CK-MB > 1.0 µkat/L also showed a high predictive accuracy (*p* < 0.01). HsTnT demonstrated the strongest diagnostic performance, with a threshold of 1231 ng/L at 18 h (AUC = 1.0; *p* < 0.01). Earlier postoperative biomarker elevations did not show significant discriminatory value. *Conclusions*: ECG remains the most accessible and fastest predictive tool for PMI detection. However, cardiac biomarkers only gain diagnostic relevance beyond 8 h postoperatively. A multimodal approach integrating biomarker kinetics and ECG changes may enhance early decision-making and improve patient outcomes, as ST-segment elevation in combination with CK-MB > 1.0 µkat/L serves as a relevant predictor. Notably, hsTnT levels > 1231 ng/L at 18 h reliably identified patients with pathological angiographic findings. These findings were derived from a highly selected cohort (~2% of all CABG patients) referred for coronary angiography and should therefore be interpreted as hypothesis-generating rather than directly generalizable to the broader surgical population.

## 1. Introduction

Perioperative myocardial infarction (PMI) is a rare but formidable complication of coronary artery bypass grafting (CABG), casting a long shadow over patient outcomes due to its association with increased mortality and morbidity [1,2,3,4]. Though the definition of PMI continues to elude consensus [5]. Consequently, reported incidence rates of PMI for isolated CABG procedures fluctuate dramatically, ranging from as low as 0.3% to as high as 30%, depending on the definition and diagnostic tools employed [3,6]. This reflects the diagnostic ambiguity and controversy surrounding PMI. The challenge lies in differentiating true ischemic myocardial injury and necrosis from the background noise of non-specific, procedure-related or postoperative changes, including surgical trauma, inflammation, and hemodynamic instability, all of which can elevate cardiac biomarkers or induce postoperative electrocardiographic alterations [7]. The diagnostic landscape for PMI has become increasingly complex, as several definitions have emerged in recent years. Among these are the Fourth Universal Definition of Myocardial Infarction (4UDMI), the Society for Cardiovascular Angiography and Interventions (SCAI) definition, and the Academic Research Consortium (ARC) definition [8,9,10]. These frameworks diverge in their enzyme cut-off thresholds, specified timing, and criteria for additional diagnostic markers, such as new electrocardiographic (ECG) abnormalities, imaging evidence of ischemia, or angiographic confirmation of bypass graft or native vessel occlusion. The European Society of Cardiology guidelines define PMI based on the Fourth Universal Definition. However, both the 4UDMI and other PMI definitions have faced growing criticism, particularly in the context of CABG. Their reliance on biomarker thresholds has been compared to navigating without a compass, uncertain whether elevations signify true ischemia or benign procedure-related phenomena. This uncertainty is compounded by the fact that the biomarker thresholds in current PMI definitions were arbitrarily established, lacking substantial evidence to support their selection at the time of introduction [7,8,9]. Adding to the complexity, the variability of these thresholds across studies further fuels diagnostic inconsistencies, making the interpretation of cardiac biomarkers feel akin to reading between the lines of a blurred manuscript. ECG changes, such as ST-segment alterations or new Q waves, offer an additional lens into ischemic processes, yet the clarity they provide is often pixelated and incomplete. The main criticism is that the inclusion of electrocardiographic findings in all current PMI definitions seems to rely more on extrapolations from data on acute coronary syndromes and coronary artery disease, as well as biological plausibility, rather than evidence derived from cardiac surgery patients [7]. Given the diagnostic ambiguities and limitations of current PMI definitions, particularly in the context of CABG, this study aims to shed light on the role of ECG changes and biomarker dynamics in identifying clinically significant coronary events. To this end, we investigated the correlation between clinically observed ECG alterations, biomarker dynamics, and angiographic findings in the context of postoperative repeat coronary angiography, with the goal of refining diagnostic strategies for PMI.

## 2. Patients and Methods

### 2.1. Patients and Study Design

**Figure 1:**
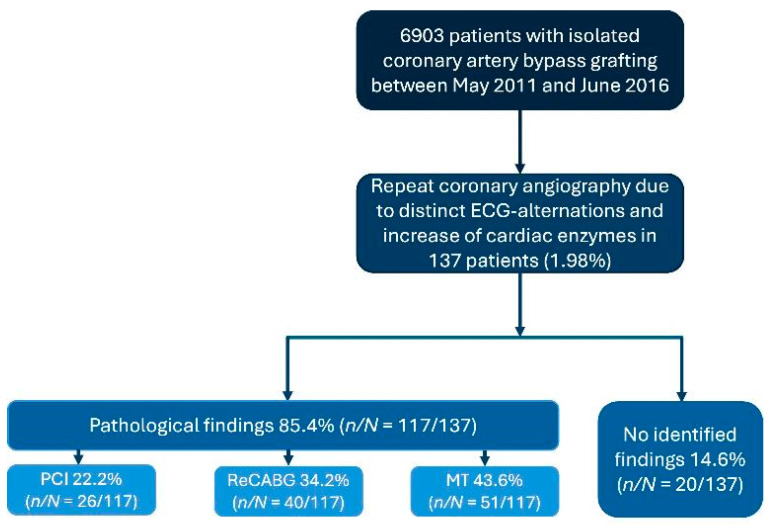
Flow diagram of the study population. Abbreviations: PCI; percutaneous coronary intervention; ReCABG, redo coronary artery bypass grafting; MT, medical treatment.

This study followed a retrospective observational design. The study included patients who underwent CABG between May 2011 and July 2016 at our institution. Among these, repeat coronary angiography was performed in 137 patients (1.98%) due to postoperative enzyme and/or ECG alterations. Pathologic angiographic findings were identified in 117 patients, while the remaining 20 patients, who exhibited ECG and enzyme alterations but no pathologic findings on repeat angiography, served as the control group (Figure 1). Preoperative, intraoperative, and postoperative data were collected, including information on potential adverse events associated with coronary angiography. Only on-pump CABG procedures were included. Off-pump CABG cases and patients with acute coronary syndrome were excluded from the analysis.

### 2.2. Decision-Making for Repeat Coronary Angiography

Creatine kinase (CK), creatine kinase myocardial band (CK-MB), the CK/CK-MB ratio (CKMBQ), and high-sensitivity Troponin T (hsTnT) as well as ECG findings were recorded immediately postoperatively. Cardiac enzyme levels were subsequently measured at 4–6 h, 8–12 h, and 18–24 h. According to our institutional protocol, pathologic findings were defined as CK levels > 10 µkat/L, CK-MB > 1 µkat/L, CKMBQ > 10%, and/or ECG alterations or relevant ventricular arrhythmias, which prompted repeat coronary angiography. Postoperative ECGs were systematically evaluated for new ischemic changes. Significant ECG abnormalities were defined as new ST-segment elevation ≥ 1.0 mm in ≥2 contiguous leads (≥2.0 mm in V2–V3 in men and ≥1.5 mm in V2–V3 in women), ST-segment depression ≥1.0 mm in ≥2 leads, new T-wave inversion ≥1.0 mm in ≥2 leads, new pathological Q-waves (≥40 ms and/or ≥25% of the R-wave), or new left bundle branch block. High-sensitive Troponin T was measured using the Roche Elecsys^®^ hsTnT assay (lower detection limit 5 ng/L; 99th percentile URL 14 ng/L) in parallel with CK and CK-MB. For clarity and consistency, time intervals in this paper are subsequently simplified to 4, 8, and 18 h to facilitate data presentation and interpretation.

### 2.3. Study Endpoints

The primary endpoint of this observational study was the identification of pathologic findings during repeat coronary angiography, irrespective of whether native vessels or CABG grafts were affected. The secondary endpoint focused on the type of treatment administered following pathologic findings, distinguishing between medical treatment (MT), percutaneous coronary intervention (PCI), and redo-CABG (ReCABG). Patients who underwent postoperative repeat coronary angiography without pathological abnormalities (no identified findings) were defined as the NIF group.

### 2.4. Data Analysis and Statistics

ECG changes and enzyme elevations were analyzed in relation to the study endpoints. Statistical analyses were conducted using SAS JMP 7.0© software and Python (version 3.9; Python Software Foundation). For continuous variables, normality was assessed using the Shapiro–Wilk test. In case of normality (*p* > 0.05), numeric variables are presented as means with standard deviation (mean ± SD), and comparisons between means were performed using Student’s *t*-test, with a significance level of *p* < 0.05. If the assumption of normality was violated (*p* < 0.05), group differences were analyzed using the non-parametric Mann–Whitney U test. Categorical data were analyzed using contingency tables and Fisher’s exact test. Following univariate analysis, a multivariate stepwise forward regression model was constructed. Additionally, a receiver operating characteristic (ROC) analysis was conducted for various enzyme values to evaluate diagnostic performance. Quality criteria, including the area under the curve (AUC), specificity, sensitivity, and likelihood ratios, were calculated. The optimal cutoff values were determined using the Youden Index. A Kaplan–Meier analysis of cumulative mortality was conducted to estimate the mortality probabilities of patients who underwent postoperative repeat coronary angiography over a maximum follow-up period of six years (2190 days). To assess mortality risk, a Cox proportional hazards model was applied, using the NIF group as the reference category. Given the limited sample size and low event rate, a bootstrap Cox regression with 1000 resamples was performed to generate robust hazard ratio (HR) estimates and corresponding 95% confidence intervals (CI, 2.5–97.5%). HRs were calculated for the MT, PCI, and ReCABG groups. The proportional hazards assumption was verified prior to analysis, and *p*-values < 0.05 were considered statistically significant. Given the exploratory character of this analysis, no formal adjustment for multiple testing was performed. Reported *p*-values are to be interpreted in a descriptive and hypothesis-generating context.

## 3. Results

### 3.1. Patient Baseline Characteristics

The mean age of the patients was 66.2 ± 8.8 years, with more than two-thirds being male (*n* = 99; 72.3%). The mean body mass index (BMI) was 27.3 ± 3.4 kg/m^2^. Chronic obstructive pulmonary disease (COPD) was present in 11 patients (8.0%), and diabetes mellitus type 2 was recorded in approximately one-third of the cohort (*n* = 42; 30.7%). A history of peripheral vascular disease was reported in 23 patients (16.8%), while cerebrovascular disease was documented in 29 patients (21.2%). Most patients exhibited three-vessel disease (*n* = 99; 72.3%), with one-vessel disease and two-vessel disease reported in only 7 (5.1%) and 31 (22.6%) patients, respectively. The left main stem (LMS) was affected in 85.4% of the patients (*n* = 117). Additionally, a history of myocardial infarction was noted in nearly half of the patients (*n* = 67; 48.9%). Detailed baseline characteristics are summarized in Table 1.

### 3.2. Procedural and Intraoperative Data

All procedures were performed using extracorporeal circulation (ECC). The mean procedure time was 162.0 ± 47.7 min, with an average aortic cross-clamp time of 41.7 ± 13.1 min. On average, 2.7 ± 0.9 anastomoses were performed per patient. The left internal mammary artery (LIMA) was utilized in 93.4% of cases (*n* = 128), while the right internal mammary artery (RIMA) was used in 5.1% (*n* = 7) (Table 2).

### 3.3. Angiographic Findings

Repeat coronary angiography was required in 137 of 6903 patients (2.0%). Pathological findings were identified in 117 cases (85.4%; per-patient analysis), while 20 patients (14.6%) showed no angiographic correlation to ECG or enzyme abnormalities (Table 3). Among those with pathological findings, medical therapy was initiated in 43.6% (*n* = 51), whereas 56.4% (*n* = 66) underwent surgical or percutaneous reintervention. The most frequently observed issues were vein graft complications (*n* = 91; 77.8%), followed by problems involving IMA grafts in 72 patients.

### 3.4. ECG Changes and Enzyme Elevations Leading to Repeat Coronary Angiography

**Figure 2:**
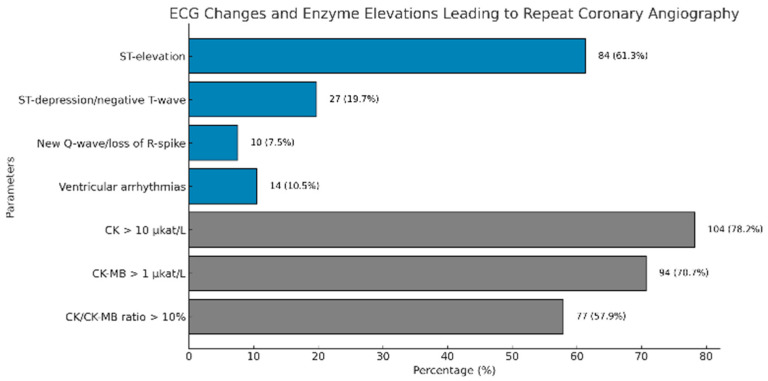
Horizontal bar chart illustrating the percentages of various ECG changes and enzyme elevations that lead to repeat coronary angiography. Enzyme-related parameters are highlighted in gray for distinction, with the percentages and corresponding sample sizes (*n*) annotated beside each bar. Abbreviations: ECG, electrocardiogram; CK, creatinine-kinase; CK-MB, creatinine- kinase myocardial band.

The most common ECG alteration observed was ST-segment elevation, occurring in 61.3% of patients. ST-segment depression or negative T-waves were noted in 19.7% of cases. New Q-waves or loss of R-wave amplitude were observed in 7.5% of patients, while ventricular arrhythmias were present in 10.5%. Regarding enzyme alterations, CK levels exceeding 10 µkat/L were elevated in 78.2% of patients, and CK-MB levels above 1 µkat/L were observed in 70.7%. The CK/CK-MB ratio was elevated (>10%) in 57.9% of cases (Figure 2, Table 4).

### 3.5. Correlation Between Angiographic Findings and ECG Changes

The most relevant ECG change was ST-segment elevation. Patients with pathologic findings in repeat coronary angiography demonstrated ST-segment elevation almost twice as frequently as those without pathologic findings (65.8% vs. 35.0%, respectively). This difference reached statistical significance in both univariate and multivariate analyses (*p* = 0.01 and *p* < 0.01, respectively). Less frequent, but still relevant, was ST-segment depression. Among patients with pathologic findings, ST-segment depression was observed in 31.6% (*n* = 37/117), compared to only 5.0% (*n* = 1/20) in patients without pathologic findings. This difference was also significant in univariate and multivariate analysis for pathological finding in the postoperative repeat coronary angiography (*p* = 0.04 and *p* = 0.02, respectively). New Q-waves, loss of R-wave amplitude, or new-onset ventricular arrhythmias were not predictive of relevant angiographic findings (Table 5: *p* > 0.999 and *p* = 0.26, respectively).

### 3.6. Correlation Between Angiographic Findings and CK-, CK-MB-, and CK/CK-MB-Excursion

In univariate analysis, CK > 10 µkat/L, CK-MB > 1 µkat/L, and the quotient CK/CK-MB > 10% were significantly associated with pathologic angiographic findings (Table 4: *p* < 0.01 for all).

### 3.7. Correlation Between Angiographic Finding and Combination of ECG Changes and CK-MB-Excursion

When analyzing the combined predictive value of ST-segment changes and CK-MB elevation, the combination of ST-segment elevation and CK-MB > 1.0 µkat/L was significantly associated with pathological angiographic findings (58.1% vs. 5.0%, *p* < 0.01) and remained a strong predictor in the univariate analysis (*p* < 0.01). In contrast, the combination of ST-segment depression and CK-MB > 1.0 µkat/L did not reach statistical significance (26.2% vs. 10.0%, *p* = 0.366) in the overall cohort, though a trend was observed in the univariate analysis (*p* = 0.09; Table 4).

### 3.8. Receiver Operating Characteristic (ROC) Analysis of Postoperative Enzyme Course of CK, CK-MB and CK/CK-MB Ratio

**Figure 3:**
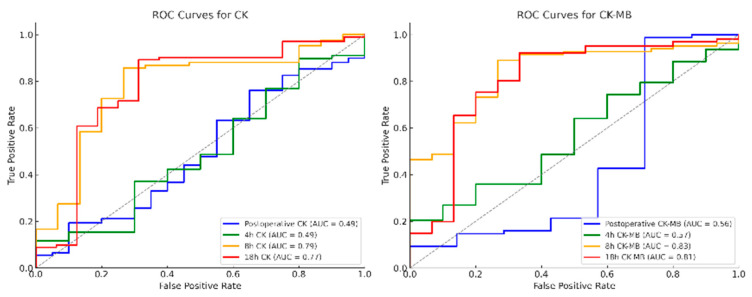
Receiver Operating Characteristic (ROC) curves for total CK and CK-MB levels measured at different timepoints after surgery (postoperative, 4 h, 8 h, and 18 h). Area under the curve (AUC) values are indicated in the legend. Abbreviations: CK, creatinine-kinase; CK-MB, creatinine-kinase myocardial band. Note: The dotted diagonal line represents the line of no discrimination (AUC = 0.5).

In ROC analysis demonstrated the statistical relevance of CK, CK-MB, and CK/CK-MB ratio excursions starting at 8 to 12 h postoperatively (Figure 3, Table 6). Cut-off values were identified for CK (8 h: 10.1 µkat/L; 18 h: 10.9 µkat/L; *p* < 0.01), CK-MB (8 h: 0.67 µkat/L; 18 h: 0.48 µkat/L; *p* < 0.01), and CK/CK-MB ratio (8 h: 6.4%; 18 h: 4.6%; *p* < 0.01). Despite AUC values ranging between 0.72 and 0.83, both specificity and sensitivity remained low (Table 5; Figure 3). Higher specificity and sensitivity were observed for changes in CK and CK-MB within the first 18 h postoperatively (∆CK18, ∆CK-MB18). A change in CK exceeding 7.04 µkat/L within 18 h postoperatively was associated with angiographic findings, showing a specificity of 77.5% and a sensitivity of 65% (AUC 0.71; *p* < 0.01). Similarly, a ∆CK-MB18 of 0.21 µkat/L achieved a sensitivity of 71% and specificity of 70% (AUC 0.77; *p* < 0.01).

### 3.9. High-Sensitivity Troponin T (hsTnT) Enzyme Course with Respect to Angiographic Findings

**Figure 4:**
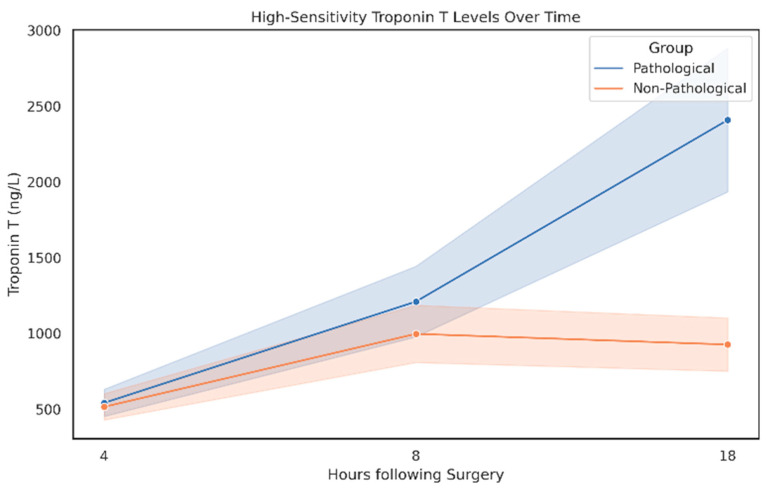
The course of high-sensitivity Troponin T over a period of 12 h.

The analysis of Troponin T levels at 4-, 8-, and 18 h post-surgery revealed a time-dependent difference between the groups. At 4 h, no statistically significant difference was observed (*p* = 0.199). However, at 8 h, a significant difference emerged (*p* < 0.001), which became highly significant by 12 h (*p* < 0.0001), with markedly higher Troponin T levels in the pathological findings group compared to the NIF group (Figure 4).

### 3.10. ROC Analysis of Postoperative Enzyme Course of hsTnT

**Figure 5:**
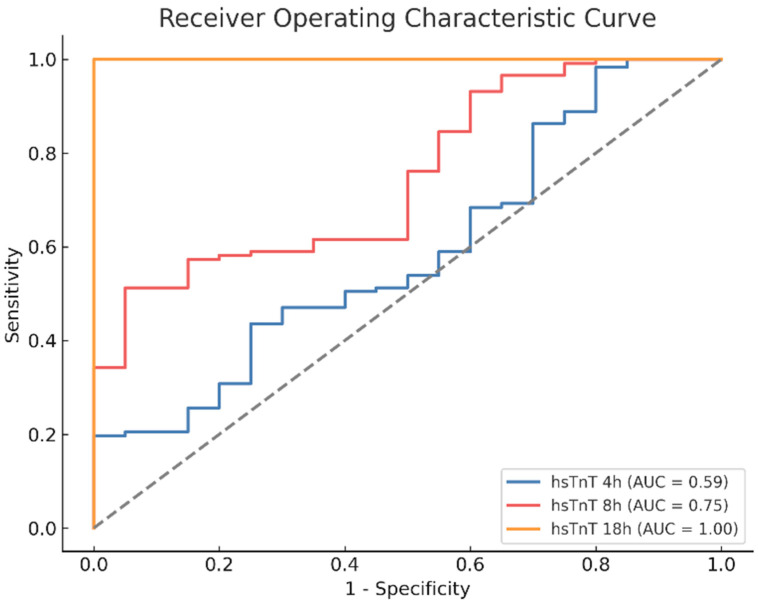
Receiver Operating Characteristic (ROC) curves suggest that high-sensitivity Troponin T (hsTnT) provides an accurate prediction of pathological findings in angiography no earlier than eight hours post-surgery. Note: The dotted diagonal line represents the line of no discrimination (AUC = 0.5).

ROC analysis was conducted to evaluate the ability of high-sensitivity Troponin T (hsTnT) levels to distinguish between pathological findings and no identified findings at different time points (4 h, 8 h, and 18 h post-surgery). At 4 h, hsTnT exhibited limited discriminatory power (AUC = 0.59), indicating poor differentiation between the groups. By 8 h, its predictive accuracy improved (AUC = 0.75), suggesting a moderate ability to distinguish between patients with and without pathological findings. At 18 h, hsTnT demonstrated near-perfect separation (AUC = 1.00). The Youden Index analysis identified the optimal cutoff values, with thresholds of 641 ng/L for troponin measurement at 4 h, 1231 ng/L at 8 h, and 1608 ng/L at 18 h. The highest diagnostic accuracy was observed for the 18 h measurement, followed by the 8 h measurement, whereas the 4 h measurement exhibited the lowest discriminatory power (Figure 5).

### 3.11. Multivariate Analysis

In the stepwise forward regression model that included both ECG and enzyme alterations with respect to CK-, CK-MB and CK/CK-MB ratio, three univariate significant factors retained their significance. ST-segment elevation and depression were identified as independent risk factors for pathologic angiographic findings (*p* < 0.01 and *p* = 0.02, respectively). ST-segment elevation in combination with CK-MB levels exceeding 1.0 µkat/L was found to be highly predictive of angiographic abnormalities (*p* < 0.001). All other factors, including cardiac enzyme elevations, did not demonstrate statistical significance (Table 5).

### 3.12. 30-Day Mortality Analysis

**Figure 6:**
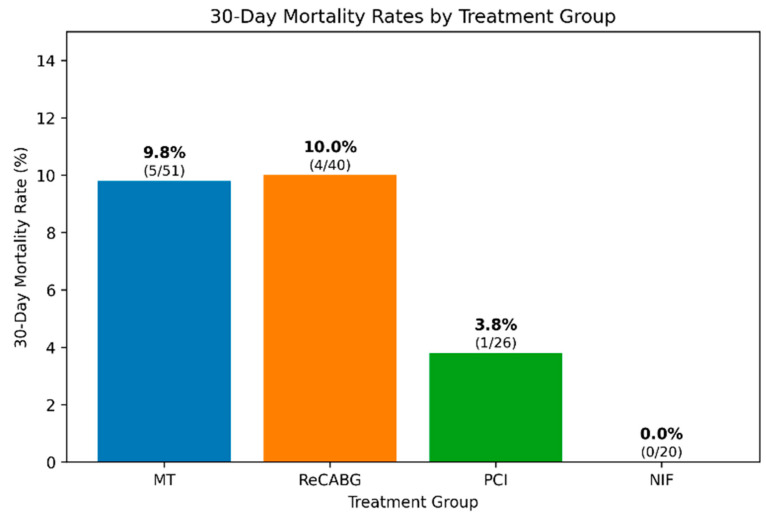
30-day mortality rates by the four different treatment group. Abbreviations: MT, medical therapy; ReCABG, redo coronary artery bypass grafting; PCI, percutaneous coronary interventions; NIF, no identified findings in the repeat coronary angiography.

Thirty-day mortality rates varied among patient groups (Figure 6). The MT and ReCABG groups had similar 30-day mortality rates of 9.8% (5/51) and 10.0% (4/40), respectively. The PCI group had a lower mortality rate of 3.8% (1/26), while no deaths occurred in the NIF group (0/20). However, no statistically significant differences in 30-day mortality were found between the groups (*p*-values ranging from 0.312 to 1.000).

### 3.13. Comparison of Long-Term Survival Across Treatment Groups

**Figure 7:**
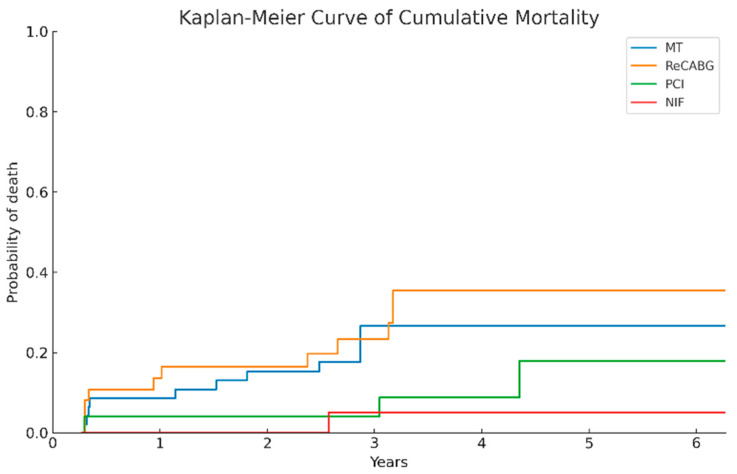
The Kaplan–Meier curve illustrate the probability of mortality over time for the four patient groups. Abbreviations: MT, medical therapy; ReCABG, redo coronary artery bypass grafting; PCI, percutaneous coronary interventions; NIF, no identified findings in the repeat coronary angiography.

The Kaplan–Meier method was applied to analyze survival probabilities over a 6-year observation period in four patient groups. Survival rates were 90.0% (45/50) in the MT group, 89.5% (34/38) in the ReCABG group, 96.0% (24/25) in the PCI group, and 95.0% (19/20) in the NIF group (Figure 7).

**Figure 8:**
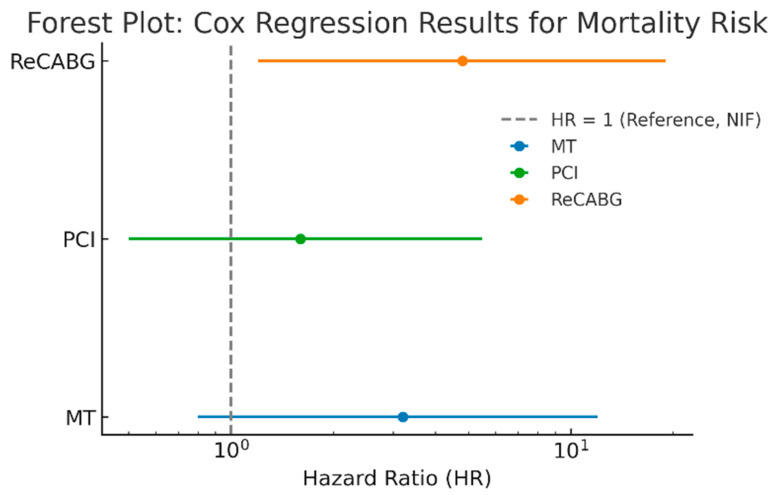
Cox regression results for mortality risk in the treatment groups. Abbreviations: MT, medical therapy; ReCABG, redo coronary artery bypass grafting; PCI, percutaneous coronary interventions; NIF, no identified findings in the repeat coronary angiography.

The bootstrap Cox regression analysis was conducted to evaluate the association between treatment groups and mortality risk, using the NIF group as the reference category (Figure 8). Given the limited sample size and low event rate, 1000 bootstrap resamples were applied to generate robust hazard ratio (HR) estimates and corresponding 95% confidence intervals (CI, 2.5–97.5%). While no statistically significant differences were observed between groups, a trend toward increased mortality risk was noted in certain treatment cohorts. Patients in the MT group exhibited a higher hazard ratio (HR = 3.2, 95% CI: 0.8–12.0, *p* = 0.172), indicating a non-significant increase in mortality risk compared to the reference group. Similarly, the PCI group showed an HR of 1.6 (95% CI: 0.5–5.5, *p* = 0.589), suggesting no statistically significant difference in mortality risk compared to the NIF group. The ReCABG group demonstrated the highest hazard ratio (HR = 4.8, 95% CI: 1.2–19.0, *p* = 0.067), reflecting a trend toward increased mortality risk, though the wide confidence interval indicates uncertainty in the estimate.

### 3.14. Adverse Events of Repeat Coronary Angiography

We recorded no procedural or vascular complications associated with repeat coronary angiography. However, additive renal impairment, characterized by an elevation in serum creatinine and the need for dialysis, occurred in 11 patients (8.0%), which was significantly higher compared to the overall CABG population (4.4%; *p* < 0.05).

## 4. Discussion

This study contributes to the ongoing debate surrounding the definition and diagnostic criteria of PMI after CABG by analyzing ECG changes and biomarker kinetics in correlation with angiographic findings. Coronary bypass surgery remains a cornerstone in the management of coronary artery disease. The diagnostic framework for acute coronary syndromes is well-established and widely accepted, offering clinicians a clear and largely standardized pathway for decision-making [11,12]. In contrast, the definition of PMI after CABG remains elusive—fragmented by heterogeneous criteria, varying biomarker thresholds, and a resulting lack of universal consensus and heterogeneity of definitions [5,13,14]. Even with a confirmed diagnosis, the optimal treatment strategy remains subject to debate, as conclusive evidence guiding decision-making is still lacking; for after the diagnosis has been established, management encompasses a spectrum of therapeutic approaches, ranging from conservative strategies to PCI and, in select cases, redo surgery [14]. However, there is clear consensus that PMI is a prognostically relevant complication, regardless of the definition used for its diagnosis [15,16]. Within the heterogeneous diagnostic landscape of PMI, postoperative repeat coronary angiography holds a pivotal role as the gold standard for identifying its underlying causes, particularly in assessing the graft patency or new coronary artery lesions [13]. Commonly available diagnostic tools such as ECG and biomarkers, which are frequently used for rapid clinical assessment and often trigger postoperative repeat coronary angiography due to presumed pathological changes, consistently exhibit a considerable overlap between patients with and without pathological angiographic findings [13,17]. This diagnostic ambiguity raises a fundamental clinical dilemma: to what extent is invasive coronary angiography truly warranted in this setting, and at what threshold does its use become indispensable for preventing secondary complications of PMI? While angiography remains the definitive tool for assessing graft patency, its routine use in the absence of clearly defined predictive markers risks unnecessary procedural burden, increased costs, and potential complications. The prevalence of postoperative repeat coronary angiography in our observed cohort of 6903 patients who underwent CABG was 2.0%, falling within the range reported in the literature (0.4–30%), with the high variability largely attributed to differences in cut-off levels defined by institutional protocols [1,18,19,20,21,22]. This highlights the need for a refined, evidence-based approach to risk stratification to ensure that invasive diagnostics are appropriately targeted.

Consistently, postoperative repeat coronary angiography identified graft-related factors as the predominant pathology (*n* = 113/117, 96.6%), while new coronary artery lesions (*n* = 4/117) were of minor significance. These findings corroborate the results of a meta-analysis and other retrospective studies, which have repeatedly highlighted graft-related complications as the principal driver of PMI [3,4,19,23,24,25].

ST-segment elevation was significantly more prevalent in patients with pathological angiographic findings compared to the control group (65.8% vs. 35.0%, *p* = 0.02) and remained a strong independent predictor in both univariate (*p* = 0.01) and multivariate analysis (*p* < 0.01). While its predictive strength was lower than ST-elevation, ST-segment depression remained a statistically significant independent predictor in both univariate (*p* = 0.04) and multivariate analysis (*p* = 0.02). By contrast, other ECG alterations, including new Q-waves, loss of R-wave amplitude, and ventricular arrhythmias, showed no significant predictive value. The lack of association may stem from the heterogeneity of postoperative cardiac electrical activity, which can be influenced by factors such as surgical trauma or hemodynamic fluctuations rather than angiographically confirmed perfusion impairments. These findings raise important questions regarding current PMI definitions, particularly concerning the role of ECG changes in different diagnostic frameworks. The SCAI explicitly includes new pathological Q-waves or persistent LBBB as diagnostic criteria alongside biomarker thresholds, while ST-segment changes are not directly emphasized [9]. However, the European Association of Cardio-Thoracic Surgery (EACTS) expert consensus statement on perioperative myocardial infarction after cardiac surgery clearly defined that an isolated newly developed Q wave has no diagnostic value in establishing PMI [13]. A statement supported by the findings of previous studies, which is also corroborated by the results of our study [13,26,27,28].

Cardiac enzyme dynamics were also evaluated, with CK > 10 µkat/L, CK-MB > 1 µkat/L, and CK/CK-MB ratio > 10% demonstrating significant correlations with pathologic angiographic findings in univariate analysis (all *p* < 0.01). However, these associations did not hold in multivariate models. This suggests that while enzyme elevations offer valuable insights into myocardial injury, their specificity in the postoperative context may be limited by the influence of nonischemic factors, such as inflammation and surgical manipulation. The ROC analysis revealed that cardiac enzymes gained diagnostic relevance only after 8 h postoperatively. The strongest predictive performance was observed for changes in CK and CK-MB within the first 18 h, with ΔCK (7.04 µkat/L) and ΔCK-MB (0.21 µkat/L) achieving moderate sensitivity and specificity. A significant increase in CK-MB in PMI has also been described in other retrospective studies, typically occurring only after a time interval of approximately 12 h [29,30]. These results highlight the importance of serial measurements rather than single time point evaluations to improve diagnostic accuracy.

Thus, our findings demonstrate that ST-segment elevation or depression—alone or in combination with CK-MB > 1.0 µkat/L—serves as a relevant predictor of graft-related complications. While current definitions such as the 4UDMI do not include ST-segment deviations as diagnostic criteria for type 5 MI, their predictive value—particularly in combination with CK-MB elevation—is supported by our data and warrants reconsideration in CABG-specific contexts.

High-sensitivity troponin T played a pivotal role in our analysis, emerging as the most robust biomarker for predicting pathological findings in postoperative repeat coronary angiography. Our ROC analysis identified a cutoff value of 1231 ng/L at 18 h postoperatively, demonstrating the strongest predictive performance for detecting graft-related complications or new coronary lesions in postoperative repeat coronary angiography. This threshold corresponds to an 88-fold increase above the upper reference limit (URL) of hsTnT, exceeding the commonly accepted PMI definitions in consensus-recommended cutoffs, including 4UDMI, ARC, and SCAI. This is consistent with recent data from NSTEMI patients undergoing PCI, where a troponin rise > 20%—as defined by current guidelines—was not prognostically relevant unless exceeding 40% and an absolute threshold of 5× URL. These findings underline the limitations of sensitivity-driven definitions and support the need for procedure-specific biomarker thresholds in settings such as CABG [31].

Beyond its diagnostic role, hsTnT has also been extensively investigated in the context of prognostic risk stratification. A recent large-scale retrospective analysis explored the relationship between postoperative troponin thresholds and mortality risk [29]. These findings further suggest that current consensus-defined cutoffs may be overly conservative in predicting adverse outcomes, as significantly higher troponin levels have been associated with increased 30-day mortality [32]. Further supporting this, the findings of Devereaux et al. demonstrated that the lowest troponin I threshold associated with increased 30-day mortality after CABG was markedly higher than those currently recommended in consensus statements. Specifically, a threshold of 5670 ng/L (218× URL) was identified as the lowest level linked to increased mortality risk [30]. This far exceeds the cutoff definitions in 4UDMI, ARC, and SCAI, raising critical questions about the appropriateness of current recommendations for CABG patients [33]. Although elevated cardiac troponin levels are clearly linked to long-term mortality, no validated threshold exists within the first 24 h post-CABG to guide further diagnostics such as coronary angiography. Yet, timely identification of angiographic pathology is critical to initiate appropriate therapy. This diagnostic uncertainty poses a clinical dilemma: overly cautious strategies may lead to unnecessary invasive procedures, while overly permissive approaches risk missing clinically significant ischemia.

In this context, our findings may help refine existing evidence by suggesting procedure-specific thresholds and diagnostic combinations—particularly the relevance of ST-segment deviations alongside CK-MB elevations occurring beyond 8 h postoperatively and a high-sensitivity troponin T cutoff of 1231 ng/L at 18 h. These parameters could improve diagnostic precision and support the development of CABG-adapted PMI criteria.

Once the diagnosis of PMI is established, the critical question arises: what is the optimal management strategy, and within what time frame should it be implemented? The overarching goal must be to minimize myocardial damage while ensuring that the time interval between diagnosis and potential reintervention—regardless of the modality—remains as short as possible. Thus, timely reintervention may aid in salvaging reversibly injured myocardium, limiting further ventricular dysfunction, and ultimately improving prognosis [22]. Our long-term survival analysis revealed the highest mortality risk in patients requiring redo-CABG, with a clear trend despite not reaching statistical significance. In contrast, PCI-treated patients in our cohort demonstrated long-term survival rates comparable to those without pathological angiographic findings. While the lack of statistical significance may be attributed to the study’s sample size, it could also suggest an emerging trend in which PCI serves as a viable revascularization strategy for select patients with graft failure or native coronary progression. Supporting this notion, Thielmann et al. reported that redo-CABG in acute PMI was associated with the highest in-hospital and 1-year mortality rates among all treatment groups, further highlighting the procedural risk and perioperative burden linked to surgical reintervention [21].

Taken together, treatment decisions should be individualized, weighing clinical presentation, biomarker trends, and angiographic findings. While redo-CABG remains the definitive option in certain scenarios, its high risk highlights the need for patient-specific strategies and further research into optimal revascularization pathways.

## 5. Conclusions

This study provides a structured analysis of ECG changes and cardiac biomarker kinetics in relation to pathological findings on repeat coronary angiography after CABG. PMI was predominantly associated with graft-related complications, underscoring the need for targeted revascularization strategies. CK, CK-MB, and hsTnT gained diagnostic relevance only beyond 8 h postoperatively, with hsTnT > 1231 ng/L at 18 h showing the strongest predictive value for angiographic pathology.

To support clinical decision-making, a structured postoperative monitoring protocol may be helpful, combining serial assessment of cardiac biomarkers at defined time points (4, 8, and 18 h) with ECG analysis focusing on ST-segment deviations. Based on these findings, a pragmatic risk stratification approach appears feasible: ST-segment elevation in combination with CK-MB > 1.0 µkat/L within the first 8 h, and particularly hsTnT levels > 1231 ng/L at 18 h postoperatively, may serve as reliable indicators to guide the indication for repeat coronary angiography. In particular, the presence of ST-segment elevation or depression in the immediate postoperative ECG should raise suspicion and warrant close attention to the dynamic course of cardiac enzymes. In this context, rising CK-MB levels (>1.0 µkat/L at 8 h) and elevated hsTnT values (>1231 ng/L at 18 h) may provide valuable diagnostic guidance. Conversely, in patients without ECG abnormalities, isolated biomarker elevations should be interpreted with caution and ideally supported by echocardiographic findings.

These observations may serve as a basis for future prospective studies aimed at evaluating the clinical utility of such thresholds. While not sufficient to directly influence current guidelines, the findings may eventually contribute to discussions in future updates of the Fourth Universal Definition of Myocardial Infarction (4UDMI) or the SCAI classification.

Long-term survival analysis revealed the highest mortality in patients requiring redo-CABG, whereas PCI-treated patients had outcomes comparable to those without angiographic pathology. Despite advancements in high-sensitivity biomarkers, ECG remains the most accessible and predictive first-line diagnostic tool for PMI. These findings highlight the need for CABG-specific thresholds and diagnostic criteria. A multimodal approach combining ECG, biomarker kinetics, and angiographic findings may enhance early decision-making and reduce unnecessary invasive procedures.

## 6. Limitations

This study has several limitations that should be acknowledged. First, its retrospective design inherently limits causal interpretations and introduces potential selection bias. Only patients who underwent postoperative coronary angiography due to clinical suspicion of PMI were included, representing a small and highly selected subset (approximately 2%) of the overall CABG population. This selection bias limits generalizability and precludes assessment of the true diagnostic performance of ECG or biomarkers in an unselected postoperative setting.

Second, the control group (patients with ECG/enzyme changes but without pathological angiographic findings) was small (*n* = 20), which reduces statistical power and may affect the robustness of between-group comparisons.

Third, although multivariate analysis and bootstrap regression were applied to strengthen robustness, residual confounding cannot be entirely excluded. The relatively small sample size, particularly in the subgroup analyses of different treatment strategies, may have contributed to the lack of statistical significance in certain comparisons, despite observed trends in mortality risk. Due to the sample size, the reliability of the ROC analysis and threshold determination should be interpreted with caution.

Fourth, while ST-segment changes and cardiac biomarkers were analyzed as diagnostic markers for angiographic pathology, their postoperative specificity is limited. ST-segment alterations can also occur due to non-ischemic factors such as pericarditis, electrolyte imbalances, or surgical trauma.

Finally, this was a single-center study with institution-specific protocols for repeat coronary angiography. Echocardiographic evaluation of regional wall motion abnormalities was not systematically performed, which could have provided functional insights into myocardial viability. Biomarker thresholds and reintervention strategies may differ across institutions, limiting external validity.

Nonetheless, our findings suggest a potential association between ST-segment changes and graft-related pathology. However, the retrospective and highly selective nature of the dataset limits the generalizability of these results. Further prospective validation in unselected CABG cohorts is warranted.

## Figures and Tables

**Table 1:** Baseline characteristics.

	Overall Cohort *n* = 137
Age (years), mean ± SD	66.2 ± 8.8
Sex (male), *n* (%)	99 (72.3)
BMI (kg/m^2^), mean ± SD	27.3 ± 3.4
Diabetes mellitus, *n* (%)	42 (30.7)
LVEF, mean ± SD	56.5 ± 11.8
Atrial fibrillation, *n* (%)	19 (13.9)
COPD, *n* (%)	11 (8.0)
Renal insufficiency, *n* (%)	18 (13.1)
PAOD, *n* (%)	23 (16.8)
Cerebrovascular disease, *n* (%)	29 (21.2)
Coronary artery disease, *n* (%) − Left main stenosis − One vessel disease − Two vessel disease − Three vessel disease	117 (85.4) 7 (5.1) 31 (22.6) 99 (72.3)
History of myocardial infarction, *n* (%)	67 (48.9)
EuroSCORE II (%), mean ± SD	2.3 ± 1.9

Abbreviations: SD, standard deviation; BMI, body mass index; LVEF, left ventricular ejection fraction; COPD, chronic obstructive pulmonary disease; PAOD, peripheral arterial occlusion disease.

**Table 2:** Procedural and intraoperative data.

	Overall Cohort *n* = 137
Procedure time (min), mean ± SD	162.0 ± 47.7
CPBT (min), mean ± SD	51.4 ± 17.3
ACCT (min), mean ± SD	41.7 ± 13.1
Number of grafts, *n* (%)	2.9 ± 0.7
Number of anastomoses, mean ± SD	2.7 ± 0.9
LIMA, *n* (%)	128 (93.4)
RIMA, *n* (%)	7 (5.1)

Abbreviations: SD, standard deviation; min, minutes; CPBT, cardiopulmonary bypass time; ACCT, aortic cross-clamp time; LIMA, left internal mammary artery, RIMA, right internal mammary artery.

**Table 3:** Angiographic findings.

	Overall Cohort *n* = 137
Pathologic angiographic findings, *n* (%)	117 (85.4)
− New coronary artery lesions, *n*/*N* (%)	4/117 (3.4)
− Graft related complications, *n*/*N* (%)	113/117 (96.6)
○ One-vessel failure, *n*/*N* (%)	76/117 (65)
○ Two-vessel failure, *n*/*N* (%)	25/117 (21.4)
○ Three- and more-vessel failure, *n*/*N* (%)	12/117 (10.3)
○ IMA graft problems, *n*/*N* (%)	72/117 (61.5)
▪ Graft occlusion, *n*/*N* (%)	36/72 (50)
▪ Graft stenosis ≥75%, *n*/*N* (%)	22/72 (30.6)
▪ Other, *n*/*N* (%)	14/72 (19.4)
○ Vein graft problems, *n*/*N* (%)	91/117 (77.8)
▪ Graft occlusion, *n*/*N* (%)	55/91 (60.4)
▪ Graft stenosis ≥75%, *n*/*N* (%)	24/91 (26.4)
▪ Other, *n*/*N* (%)	12/91 (13.2)
Angiographic findings needing re-intervention (PCI or CABG), *n* (%) − PCI, *n* (%) − CABG, *n* (%)	66 (47.4) 26 (19.0) 40 (29.2)
Pathologic angiographic findings treated conservatively, *n* (%)	51 (37.2)
No pathologic angiographic findings, *n* (%)	20 (14.6)
LIMA, *n* (%)	128 (93.4)
RIMA, *n* (%)	7 (5.1)

Abbreviations: SD, standard deviation; min, minutes; IMA, internal mammary artery, LIMA, left internal mammary artery, RIMA, right internal mammary artery.

**Table 4:** ECG changes and enzyme elevations leading to repeat coronary angiography.

	Overall Cohort *n* = 137
ECG changes	
− ST-elevation, *n* (%)	84 (61.3)
− ST-depression/negative T-wave, *n* (%)	27 (19.7)
− New Q-wave/loss of R-spike, *n* (%)	10 (7.5)
− Ventricular arrhythmias, *n* (%)	14 (10.5)
Enzyme elevations	
− CK > 10 µkat/L, *n* (%)	104 (78.2)
− CK-MB > 1 µkat/L, *n* (%)	94 (70.7)
− CK/CK-MB ratio > 10%, *n* (%)	77 (57.9)

Abbreviations: ECG, electrocardiogram; CK, creatinine-kinase; CK-MB, creatinine-kinase myocardial band.

**Table 5:** ECG changes and enzyme cutoff values with respect to angiographic findings.

	Overall Cohort *n* = 137				
	Pathological Findings *n* = 117	No Identified Finding *n* = 20	*p*-Value	Univariate Analysis *p*-Value	Multivariate Analysis *p*-Value
ECG changes					
− ST-elevation, *n* (%)	77 (65.8)	7 (35.0)	0.02 *	0.01 *	<0.01 *
− ST-depression, *n* (%)	37 (31.6)	1 (5.0)	0.01 *	0.04 *	0.02 *
− New Q-wave/loss of R-spike, *n* (%)	9 (7.7)	1 (5.0)	>0.999	>0.999	0.82
− Ventricular arrhythmias, *n* (%)	11 (9.4)	3 (15.0)	0.716	0.43	0.26
Enzyme elevation					
− CK > 10 µkat/L, *n* (%)	94 (80.3)	10 (50.0)	<0.01 *	<0.01 *	0.15
− CK-MB > 1 µkat/L, *n* (%)	86 (73.5)	8 (40.0)	<0.01 *	<0.01 *	0.36
− CK/CK-MB ration > 10%, *n* (%)	72 (61.5)	5 (25.0)	<0.01 *	<0.01 *	0.17
Combination					
− ST-elevation and CK-MB > 1 µkat/L, *n* (%)	68 (58.1)	1 (5.0)	<0.01 *	<0.01 *	<0.001 *
− ST-depression and CK-MB > 1 µkat/L, *n* (%)	26 (22.2)	2 (10.0)	0.366	0.09	0.07

Abbreviations: ECG, electrocardiogram; CK, creatinine-kinase; CK-MB, creatinine-kinase myocardial band. Note: *, *p* < 0.05.

**Table 6:** ROC analysis of postoperative enzyme course.

	**CK**					
**Time course**	**Cut-off point**	**AUC**	**Sensitivity**	**1-Specifity**	**LR^+^/LR^−^**	***p*** **-value**
**Postoperative**	3.0	0.49	75.7%	35.0%	1.2/0.7	0.78
4 h	24.4	0.49	20.5%	0.0%	∞ ^†^/0.9	0.54
8 h	10.1	0.79	89.2%	73.3%	3.2/0.2	*<0.01* *
18 h	10.9	0.77	92.1%	68.7%	2.9/0.2	*<0.01* *
	**CK-MB**					
**Time course**	**Cut-off point**	**AUC**	**Sensitivity**	**1-Specifity**	**LR^+^/LR^−^**	***p*** **-value**
**Postoperative**	0.9	0.56	77.6%	57.1%	1.4/0.1	0.90
4 h	1.6	0.57	20.5%	100.0%	∞ ^†^/0.8	0.24
8 h	0.7	0.83	89.2%	73.3%	3.3/0.1	*<0.01* *
18 h	0.5	0.81	92.1%	67.7%	2.8/0.2	*<0.01* *
	**CK/CK-MB ratio**					
**Time course**	**Cut-off point**	**AUC**	**Sensitivity**	**1-Specifity**	**LR^+^/LR^−^**	***p*** **-value**
**Postoperative**	6.6	0.59	84.0%	57.1%	1.5/0.4	0.45
4 h	6.6	0.61	69.2%	40.0%	1.7/0.5	0.27
8 h	6.4	0.72	69.5%	26.7%	2.6/0.4	*0.01* *
18 h	4.6	0.78	84.2%	33.3%	2.5/0.2	*<0.01* *
	**∆CK18, ∆CK-MB18**					
**Time course**	**Cut-off point**	**AUC**	**Sensitivity**	**1-Specifity**	**R^+^/LR^−^**	***p*** **-value**
∆CK18	7.0	0.7	77.5%	35.0%	2.2/0.4	*<0.01* *
∆CK-MB18	0.2	0.77	79.7%	30.0%	2.7/0.3	*<0.01* *

Note: Bold and italic values indicate statistical significance: *, *p* ≤ 0.05; Abbreviations: ROC, receiver operating characteristic; AUC, area under the curve; CK, creatinine-kinase; CK-MB, creatinine-kinase myocardial band; ∆CK18, changes in creatinine-kinase within the first 18 h postoperatively; ∆CK-MB18; changes in creatinine-kinase myocardial band within the first 18 h postoperatively; LR^+^, positive likelihood ratio; LR^−^, negative likelihood ratio. Note: *, *p* < 0.05; ^†^, LR+ is undefined due to perfect specificity (dividing by zero); in practice this indicates no false positives observed in this sample.

## Data Availability

The data presented in this study are available on request from the corresponding authors. The data are not publicly available due to ethical regulations.
